# Can ChatGPT be trusted? Evaluating AI responses to oral health questions among pregnant Arabic-speaking women

**DOI:** 10.1186/s12903-025-06909-z

**Published:** 2025-10-10

**Authors:** Khalid Talal Aboalshamat, Jomanh Humied Alnafei, Lojain Ahmed Alkhattabi, Ghadi Yaqoub Alhawsawi, Shrooq Majed Alahmadi, Shatha Omar Almalki, Afnan Anas Nassar

**Affiliations:** 1 Dental Public Health Division, Preventative Dentistry Department, College of Dental Medicine, Umm Al-Qura University, Makkah, Saudi Arabia; 2 College of Dental Medicine, Umm Al-Qura University, Makkah, Saudi Arabia

**Keywords:** Oral health information, Pregnancy, ChatGPT, Artificial intelligence, Machine learning, Arabic

## Abstract

**Background:**

ChatGPT, an artificial intelligence (AI) chatbot developed by OpenAI, is increasingly being used in healthcare, including dentistry, for patient education; this study aimed to assess the usability and quality of ChatGPT’s responses to pregnancy-related oral health queries in Saudi Arabia.

**Method:**

This two-part cross-sectional study assessed pregnant Arabic women’s perceptions of ChatGPT for oral health queries and evaluated its responses using an online questionnaire. Responses from ChatGPT-4o mini were rated by 5 dental experts with regard to accuracy, clarity, relevance, and acceptance using a 5-point Likert scale.

**Results:**

Among the 300 participants, 42.0% (126) knew about ChatGPT, 33.7% (101) had previously used it, 14.3% (43) had used it to obtain medical information, 8.7% (26) had used it for dental information, and 8.3% (25) had used it for dental information during pregnancy. Attitudes regarding ChatGPT were rated from 1 to 4. Except for 1 item, the means were all above the midpoint. Attitude ratings ranged from a mean of 2.71 (SD 0.76) for ChatGPT competency to a mean of 2.34 (SD 0.92) for its ability to replace human interactions. However, ChatGPT competency (*P* = .028), security (*P* = .015), willingness to use ChatGPT for inquiries (*P* = .021), ability to assist in informed decision-making (*P* = .01), willingness to make decisions based on recommendations (*P* = .024), and persuasiveness (*P* = .049) were significantly different based on educational level. Pregnant women with higher levels of education rated these aspects significantly lower than those with a high school diploma or bachelor’s degree.

**Conclusion:**

ChatGPT provided useful oral health information for pregnant individuals, but its responses required revision and supervision by health professionals. Its usage among pregnant women in Saudi Arabia remained low.

**Supplementary Information:**

The online version contains supplementary material available at 10.1186/s12903-025-06909-z.

## Background

ChatGPT, developed by OpenAI, is a sophisticated language model capable of generating human-like responses to even complex questions utilizing artificial intelligence (AI) [1]. Machine learning (ML), a subset of AI, allows computer systems to analyze data, recognize patterns, and make decisions without the need for specific programming [2]. A key example of ML is a large language model (LLM), such as ChatGPT, which powers chatbot functions in natural language processing (NLP) to interpret and respond to human language in a conversational style [3]. AI is becoming crucial in healthcare in order to cope with rising clinical demands that lead to longer patient wait times, increased burnout, and strained resources [4]. One example is the use of chatbots such as ChatGPT to enhance patient understanding by answering their queries about treatments, complications, and prognosis [5, 6].

AI is proving valuable in dentistry by improving treatment. For instance, AI showed greater sensitivity at detecting early caries than dentists, enabling noninvasive treatment and avoiding costly procedures [7, 8]. Studies also suggest that AI can enhance diagnostic skills and reduce time spent on treatment planning [9].

Several studies have tried to assess ChatGPT as a source of dental information. Some of these studies assessed the accuracy of ChatGPT as a provider of dental information in different areas, such as head and neck, oromaxillofacial surgery, endodontics, and orthodontics [10–13]. The accuracy ranged between 58% and 95% [10–13]. This might be attributed to the quality and clarity of user input given that it determines ChatGPT’s answers. Inaccurate or irrelevant responses might result from ambiguous or incomplete questions [14]. Other studies have assessed the level of comprehensiveness, revealing a variation between open-ended questions (73.0%) and clinical case scenarios (56.7%) [10]. ChatGPT answers were found to be 73.3% logical and 93.3% comprehensiveness regarding children’s oral health [15].

Other studies have attempted to compare different models of AI chatbots. For example, one study compared ChatGPT-3.5, Google Bard, and Bing with questions regarding endodontics [13]. On a high threshold validity test, ChatGPT was the most credible (60%) compared to Google Bard (15%) and Bing (15%) [13]. The difference in the responses of these AI chatbots can be attributed to the varying design philosophies of their companies, including the datasets used for training, the objectives that they are designed to achieve, and the specific algorithms employed [16, 17]. Another study compared an embedded GPT model (trained on specific provided information and base prompt [18]) to ChatGPT with regard to different dental domains [18]. The embedded model showed superior performance with accuracy of 62.5%, compared to ChatGPT’s responses with 52.5% accuracy [18]. This indicated that AI chatbots can generate different results based on the model of the setting.

Most of the previous studies tried to assess the accuracy of ChatGPT answers based on questions given by dental professionals [10–13]; however, one study evaluated the answers based on questions asked by patients [19]. This might give some variability because answers to dental specialists might not be suitable for the general public, and vice versa [14].

It has been reported that the use of AI as a source of information in health fields has some hazards. For example, Google Bard recommended that pregnant patients with allergies to local anesthetics should undergo general anesthesia for a root canal treatment in order to avoid complications, demonstrating a limitation in handling complex queries [13]. Also, ChatGPT did not provide references to various clinical case scenarios in 46.4% of cases [10]. In fact, researchers recommended that ChatGPT should not be used without professional supervision [20]. Thus, AI chatbots could not take the place of a dentist [21], at least not in the current format.

All of the previous studies used questions to ChatGPT in English. There has been no single study that assessed performance when the user spoke Arabic. This is important because the Arabic language is considered the fifth most widely spoken language in the world, with over 422 million individuals who speak it and 22 countries using Arabic as their official language [22].

Despite the number of dental topics that have been used in the assessment of the accuracy of ChatGPT, some important topics have not been thoroughly investigated, such as oral health in pregnant women. In fact, a woman’s oral health can be adversely affected by pregnancy gingivitis, pregnancy granuloma, caries, xerostomia, and tooth mobility, among other complex physiological changes affecting oral health [23]. Pregnant women have been observed to seek health information during the perinatal period, throughout pregnancy, and after birth [24]. However, in terms of dental health, many pregnant women tend to avoid seeing a dentist, which can be a source of medical information and therapy [25]. This shows how important it is to increase pregnant women’s awareness of oral health and pregnancy-related dental care issues [26].

In addition to the prior gaps in research, there has been no study assessing the usability of ChatGPT as a source of oral health information for pregnant women. Therefore, our study aimed to assess the usability and quality of ChatGPT’s responses to questions related to oral health during pregnancy in Saudi Arabia.

## Methods

### Part 1

#### Participants

A convenience sampling method was used to recruit currently pregnant women or women who were pregnant (married or divorced) within the past year who were aged 18 years or older and residing in Saudi Arabia. The exclusion criteria were those women who had no access to the internet and those who could not read or write. Based on an estimated prevalence of 50%, a margin of error (precision) of 5%, and a 90% confidence level, the minimum sample size required for this study was calculated to be 271 participants. The calculation was based on the following equation: (n = (Z² × P × (1 − P))/d²), where Z is the Z-score corresponding to the desired confidence level (1.645), P is the estimated prevalence (50%), d is the desired precision (0.05). Participants in Part 1 were recruited via an online survey distributed through various social media platforms, including WhatsApp, Twitter, Snapchat, Telegram, and Instagram. The research team sent invitations to potential participants’ personal accounts and pregnant women’s groups on social media. Additionally, personal invitations to the study were sent out by the research team. The study was approved by the institutional review board of Umm Al-Qura University, Makkah, Saudi Arabia, with the number of HAPO-02-K-012-2024-10-2317. The questionnaire was administered anonymously, and any information that could potentially reveal participants’ identities was removed prior to analysis.

#### The questionnaire

The study questionnaire was provided to participants in electronic format in Arabic. To protect participants’ confidentiality, each questionnaire had an assigned identification number, ensuring anonymity. Informed consent was obtained electronically by requiring participants to click an “approve” button before accessing the questionnaire. Those who did not agree were considered to have declined consent and were excluded from the study.

The questionnaire comprised 28 questions divided into 3 sections. Section 1 included 13 demographic questions about age, nationality, region of residence, marital status, pregnancy status, number of children, educational level, sources of oral health information, familiarity with ChatGPT, and previous experience using ChatGPT for general health information and for pregnancy-related dental information.

**Fig. 1:**
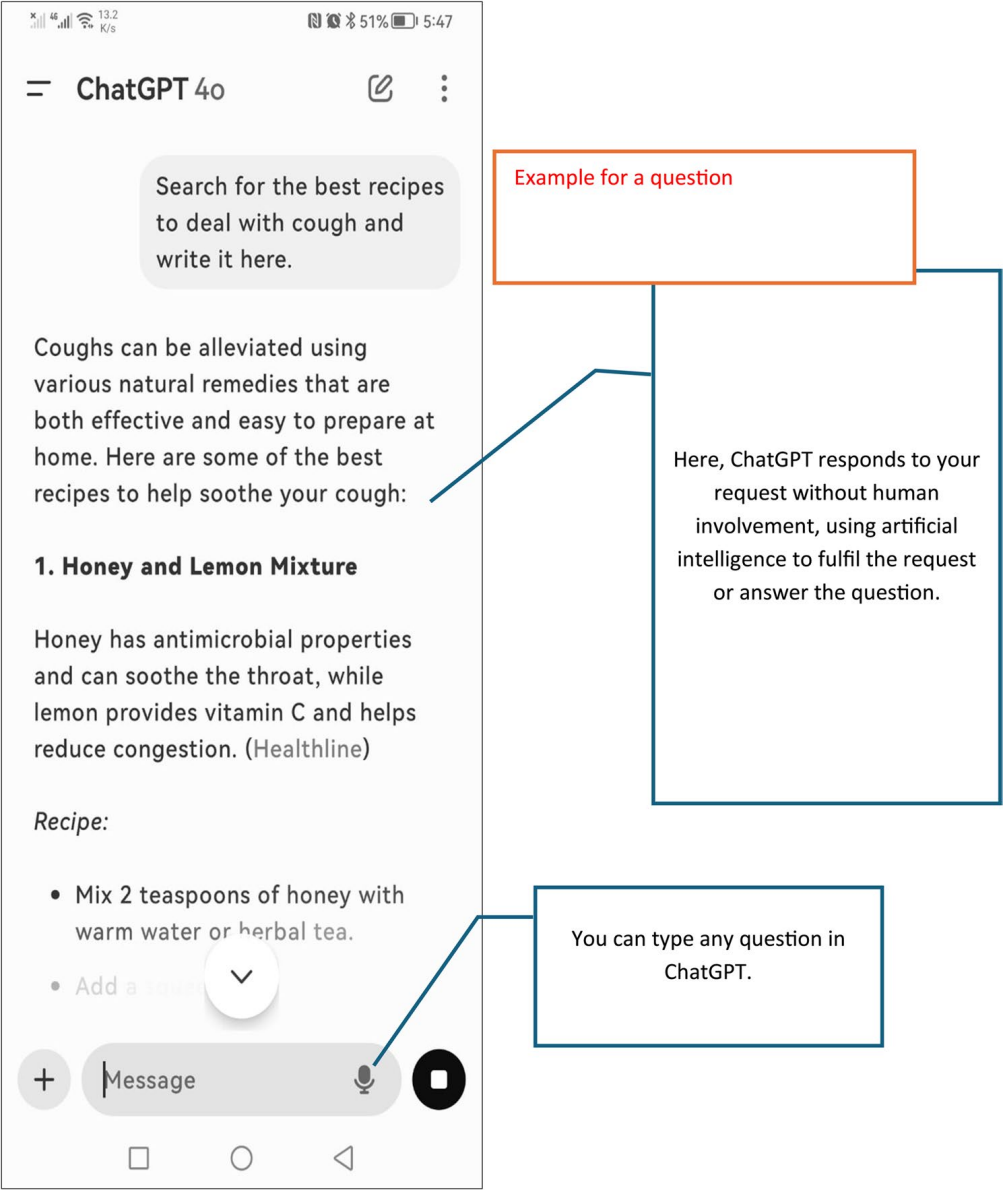
Explaining ChatGPT to participants (English version)

Since some participants did not know about ChatGPT before, ChatGPT was explained to the participants in the beginning of Sect. 2 in text by stating “If you are not familiar with ChatGPT, we would like to introduce it to you. ChatGPT is an intelligent program that uses artificial intelligence to communicate with you, either through text or voice, and to answer your questions using information available on the internet. It functions like a personal assistant that understands what you say and provides answers or advice based on what it has learned.” This information was given in Arabic. Also, ChatGPT was explained by a picture, Fig. 1. The participants were asked not to go and change their answer**s** in Sect. 1 based on this new information.

Section 2 consisted of 14 questions adapted from a previous study [27], modified to focus on the use of ChatGPT for pregnancy-related oral health. These questions, originally derived from the Unified Theory of Acceptance and Use of Technology (UTAUT) [28], assessed ChatGPT’s competence, reliability, transparency, trustworthiness, security, response integrity, decision-making support, persuasiveness, and the human’s willingness to use it for healthcare queries, perception of benefits, satisfaction with the model, future use potential, and potential to replace human interaction. Responses were recorded using a 4-point Likert scale (1, strongly disagree, to 4, strongly agree). A pilot test of 12 participants was previously conducted to confirm the validity of the content regarding language, syntax, logic, coherence, and comprehension. Section 3 included a single open-ended question that encouraged participants to submit as many oral-health-related queries as they wished concerning pregnancy.

### Part 2

#### Question selection process: source, exclusions, and inclusions

A set of pregnancy-related oral health questions was collected in Arabic from the open-ended responses provided by pregnant women in Part 1 of the questionnaire. Initially, 124 questions were gathered. All questions directly related to pregnancy and oral health were retained, while duplicates, questions pertaining to general health, questions written in wrong text in Arabic, or unrelated topics were excluded. After screening and removal of redundancies, a total of 50 questions remained, covering topics such as prevention (6 questions), anesthesia and tooth extraction (5), pain management (4), safety and radiographic procedures (5), medications and supplements (8), toothpaste, mouthwash, fluoride, and bleaching (9), periodontology, orthodontics, endodontics, and dental caries (7), dental problems during pregnancy and lactation (3), and halitosis (3). Measures were taken to maximize the diversity of questions to address common concerns regarding oral health during pregnancy.

#### ChatGPT setup and procedure

ChatGPT-4o mini (free version) was used to process the questions. A new email address was created to minimize the impact of search algorithm biases. Access to ChatGPT-4o mini was initiated via a Google search, selecting the “Continue with Google” option during login. Google was chosen to simulate a typical user experience because it is the most widely used search engine.

Each question was submitted to ChatGPT-4o mini by a designated team member, and the corresponding responses were compiled into a Word document. Due to the large number of questions and the daily query limitations imposed by the platform, data collection was conducted over six consecutive days. To ensure consistency, all queries were submitted at the same time each day (7:00 PM) using the same computer and network environment.

Before querying ChatGPT-4o mini, all browsing history and cookies were cleared using the Clear Browsing Data feature in the Privacy and Security settings. Options such as Cached Images and Files, Cookies and Other Site Data, and Browsing History were selected with an appropriate time frame to meet the study’s requirements.

#### Quality assessment of ChatGPT-4o mini answers

Each ChatGPT-4o mini’s response to pregnancy-related oral health questions were evaluated by 5 senior dental students in the final years (6th year dental students, nearly graduated dentists) supervised by a professor in dental public health. A 5-point Likert scale (1, strongly disagree, to 5, strongly agree) was used to assess responses based on a previous study [19], considering the following criteria; (1) accuracy: whether the response was correct according to literature and dental scientific guidelines, (2) clarity: whether the answer was easy to understand, (3) comprehensiveness: whether the response covered all aspects of the query, (4) relevance: whether the response directly addressed the question, and (5) acceptance: whether the response was acceptable without modifications.

Prior to evaluation, the 5 assessors were calibrated using a set of ten questions to standardize their assessments of each ChatGPT-4o mini response. Variations in the length and depth of the responses were observed; however, each sentence was assessed. Each information was classified into: commonly known by general dentist, questionable, or seemingly incorrect information. Any information identified as questionable or potentially incorrect by any assessor was color-coded for further discussion. These discussions, conducted under the guidance and supervision of the professor, aimed to achieve consensus on the final evaluation. The determination of accuracy for such content was ultimately based on supporting evidence from established dental literature and clinical guidelines. In instances where sentences contradicted evidence-based findings, the content was subjected to further discussion by the research team.

#### Data analysis for part 1 and part 2

Statistical analysis was performed using SPSS software version 29 (IBM Corp., Armonk, NY, USA). In addition to descriptive statistical methods, a t-test was used to compare data variables. The results were evaluated at a significance level of *P* <.05.

## Results

In this study, 300 pregnant women completed the study questionnaire. Participant age was a mean (m) of 29.02 years with a standard deviation (SD) of 6.19. Participants had a mean of 1.64 children (SD 1.59). Participant demographic data are shown in Table 1.

**Table 1:** Participant demographic data

Variables		*n* (%)
Nationality	Saudi	260 (86.7)
	Non-Saudi	40 (13.3)
Region	Western	162 (54.0)
	Central	58 (19.3)
	Eastern	32 (10.7)
	Southern	33 (11.0)
	Northern	15 (5.0)
Marital status	Married	297 (99.0)
	Not married currently (divorced)	3 (1.0)
Pregnancy status	Currently pregnant	159 (53.0)
	Pregnant within last year	141 (47.0)
Education	High school or lower	63 (21.0)
	Bachelor’s degree	216 (72.0)
	Higher education	21 (7.0)

The pregnant women had different sources of information regarding oral health, as displayed in Table 2.

**Table 2:** Pregnant women’s source of information regarding oral health in Saudi Arabia

Variable	Yes, *n* (%)
Dentist	289 (96.3)
Social media	230 (76.7)
Internet	228 (76.0)
Family and friends	182 (60.7)
Medical doctor	99 (33.0)
Books	83 (27.7)
TV	67 (22.3)
ChatGPT	46 (15.3)
Others	80 (26.7)

Regarding the usability of ChatGPT, the participants had different responses, as shown in Table 3.

**Table 3:** Pregnant women’s view of the usability of ChatGPT in Saudi Arabia

Question	Yes *n* (%)
Do you know what ChatGPT is?	126 (42.0)
Have you ever used ChatGPT in general?	101 (33.7)
Have you ever used ChatGPT to get medical information?	43 (14.3)
Have you ever used ChatGPT to get dental information?	26 (8.7)
Have you ever used ChatGPT to get dental information during pregnancy?	25 (8.3)

Chi-square and Fisher’s exact test showed there was no significant difference when we tested the previous 5 questions regarding usability of ChatGPT, in Table 3, according to nationality, region, marital status, and pregnancy status. However, a Chi-square test showed a significant association between education level and knowing what ChatGPT is (*P =*.004). Awareness of ChatGPT increased with higher education levels, with 25.4% (16/63) of participants with a high school education or less, 44.9% (96/216) with a bachelor’s degree, and 61.9% (13/21) with higher education reporting familiarity. Nevertheless, there was no significant difference in the other 4 questions with the educational variable.

Using logistic regression, age was a significant predictor of knowing ChatGPT (*P =*.036; Exp(B) = 1.042; CL:1.003, 1.083), indicating that each additional year of age increased the odds by 4.2%. Similarly, age was significantly associated with using ChatGPT (*P =*.024; Exp(B) = 1.049; CL; 1.006, 1.093), with each additional year increasing the odds by 4.9%. The models explained 2.0% and 2.4% of the variance, respectively, as indicated by Nagelkerke *R*². This indicates a very low variance explained by age. The other questions (3, 4, and 5) were not significantly related to age. Also, the number of children was not statistically significant with any of the 5 questions about usability of ChatGPT.

The pregnant women completed 14 attitude statements about using ChatGPT for oral health related to pregnancy, as shown in Table 4.

**Table 4:** Pregnant women’s attitudes about using ChatGPT for oral health related to pregnancy

No.	Statement	Disagree & Strongly Disagree, *n* (%)	Agree & Strongly Agree, *n* (%)	m (SD)
1	ChatGPT is competent with providing information and guidance related to oral health during pregnancy.	83 (27.7)	217 (72.3)	2.71 (0.76)
2	ChatGPT is reliable and consistent in offering dependable information about oral health during pregnancy.	112 (37.3)	188 (62.7)	2.61 (0.81)
3	ChatGPT is transparent in providing information about oral health during pregnancy.	95 (31.7)	205 (68.3)	2.67 (0.80)
4	ChatGPT is trustworthy when answering questions about oral health during pregnancy.	123 (41.0)	177 (59.0)	2.55 (0.81)
5	ChatGPT does not manipulate its responses to questions related to oral health during pregnancy.	129 (43.0)	171 (57.0)	2.54 (0.84)
6	ChatGPT is secure and protects my privacy and confidential information when answering my questions about oral health during pregnancy.	101 (33.7)	199 (66.3)	2.67 (0.89)
7	I am willing to use ChatGPT for inquiries related to oral health during pregnancy.	91 (30.3)	209 (69.7)	2.72 (0.85)
8	The benefits of using ChatGPT outweigh any potential risks associated with pregnancy inquiries.	111 (37.0)	189 (63.0)	2.60 (0.82)
9	ChatGPT helps me make informed and timely decisions about oral health during pregnancy.	114 (38.0)	186 (62.0)	2.58 (0.82)
10	I am willing to make decisions based on the recommendations provided by ChatGPT regarding oral health during pregnancy.	124 (41.3)	176 (58.7)	2.55 (0.84)
11	I am satisfied with ChatGPT as a source of oral health pregnancy-related assistance.	101 (33.7)	199 (66.3)	2.64 (0.80)
12	I am willing to use ChatGPT in the future regarding oral health during pregnancy.	98 (32.7)	202 (67.3)	2.64 (0.83)
13	ChatGPT can replace human interaction for oral health inquiries during pregnancy.	151 (50.3)	149 (49.7)	2.34 (0.92)
14	ChatGPT is persuasive when it comes to providing answers about oral health during pregnancy.	95 (31.7)	205 (68.3)	2.65 (0.83)

The use of *t* test, Mann–Whitney test, and ANOVA showed that none of the 14 items on the usability questionnaire were significantly different when compared against nationality, marital status, pregnancy status, or region. However, with ANOVA, the education level was found to be significantly different for statements 1, 6, 7, 9, 10, and 14, as shown in Table 5.

**Table 5:** Significant attitude statements about ChatGPT usage for oral care during pregnancy by educational level

item	Statement	High school or less m (SD)	Bachelor’s m (SD)	Higher education m (SD)	*P*-value (ANOVA)	Significant Differences (Tukey Post Hoc)
1	ChatGPT is competent in providing information and guidance related to oral health during pregnancy.	2.84 (0.65)	2.71 (0.77)	2.33 (0.86)	0.028	• HE < HS (*P* =.021) • No difference: Bachelor vs. others
4	ChatGPT is secure and protects my privacy and confidential information when answering my questions about oral health during pregnancy.	2.84 (0.70)	2.67 (0.93)	2.19 (0.87)	0.015	• HE < HS (*P* =.010) • No difference: HS vs. Bachelor
6	I am willing to use ChatGPT for inquiries related to oral health during pregnancy.	2.81 (0.84)	2.75 (0.84)	2.24 (0.83)	0.021	• HE < HS (*P* =.020) • HE < Bachelor (*P* =.024) • No difference: HS vs. Bachelor
9	ChatGPT helps me make informed and timely decisions about oral health during pregnancy.	2.71 (0.73)	2.59 (0.82)	2.10 (0.83)	0.01	• HE < HS (*P* =.007) • HE < Bachelor (*P* =.020) • No difference: HS vs. Bachelor
10	I am willing to make decisions based on the recommendations provided by ChatGPT regarding oral health during pregnancy.	2.67 (0.78)	2.56 (0.84)	2.10 (0.83)	0.024	• HE < HS (*P* =.018) • HE < Bachelor (*P* =.042) • No difference: HS vs. Bachelor
14	ChatGPT is persuasive when it comes to providing answers about oral health during pregnancy.	2.79 (0.81)	2.64 (0.82)	2.29 (0.85)	0.049	• HE < HS (*P* =.039) • No difference: Bachelor vs. others

HS: High school or less, HE: higher education.

The content of the ChatGPT answers were evaluated by five evaluators, and the mean scores and standard deviations were as follows: accuracy (m = 4.67, SD = 0.39); clarity (m = 4.95, SD = 0.10); comprehensiveness (m = 4.87, SD = 0.20); relevance (m = 4.90, SD = 0.21); and acceptance (Mm 4.78, SD = 0.27).

## Discussion

This study assessed user perceptions of ChatGPT in addressing oral health questions during pregnancy in Saudi Arabia. The responses were rated highly in terms of accuracy, clarity, and relevance. Most participants had not previously used such tools for dental information. Usability perceptions were consistent across demographic groups, though participants with higher education levels expressed slightly less favorable attitudes.

### Part 1: quality of information

Our results showed that ChatGPT-4o mini gave relatively highly accurate, clear, comprehensive, relevant, and acceptable answers to questions related to oral health in pregnancy that were asked by the public. Our results are supported by a previous study [19] that investigated ChatGPT inquiries about oral health in India. In fact, the means of the accuracy, clarity, comprehensiveness, relevance, and acceptability in the previous study [19] were similar to our findings. Other studies have also concluded that ChatGPT was accurate, comprehensive, clear, and relevant with regard to other dental topics [10, 18, 20]. Nevertheless, some of these studies involved other populations, including dental professionals [10, 18]. Also, these previous studies investigated different dental topics and used different scales to measure the accuracy, comprehension, clarity, and relevance [10, 18, 20]. For example, one of the previous studies investigated the accuracy and clarity of ChatGPT generated information about head and neck oromaxillofacial surgery asked by professionals, using a 6-point Likert scale and a 3-point Likert scale to measure comprehensiveness [10]. Also, another study evaluated the performance of two different modes of GPT models in providing postoperative dental instructions to patients [18]. Both models were good, but one performed better than the other. This notion can be generalized more in different health domains, as other studies found ChatGPT’s accuracy in facilitating knowledge acquisition to be reliable across different domains, such as microbiology [29] and problem solving in pathology [30]. It should be noted that our study and the previously mentioned studies used different ChatGPT models, including 4o mini, 4o, 3.5, and 3.5 Turbo.

Although ChatGPT-4o mini generally delivers accurate information, some inaccuracies have been observed. ChatGPT-4o mini answered one of our questions indicating that calcium is taken up from the mother’s teeth and bones, which causes tooth fragility and cavities for the mother. That is incorrect; the fetus receives the calcium needed from the mother’s diet, not from her bones or teeth [31, 32]. Additionally, ChatGPT-4o mini answered one of the questions by stating “Breastfeeding may also contribute to calcium deficiency, which can affect dental health and increase dental pain.” Again, this is incorrect information, as during breastfeeding, calcium is absorbed from breast milk rather than the teeth, and only in small amounts from the bone, and this is temporary until weaning [33, 34].

Conversely, some of the information given was controversial in the literature. For example, ChatGPT-4o mini gave answers to one of our questions indicating that pregnant women can safely undergo dental treatment under general anesthesia during the second trimester. In fact, this information might be misleading. Despite some resources indicating that it is safe [35], research has also indicated that general anesthesia should usually be avoided during pregnancy due to potential risks to fetal brain development, including impaired learning, memory deficits, and neuronal damage, especially when exposure is prolonged or the anesthesia is administered in high doses [36]. Furthermore, ChatGPT-4o mini suggested that an implant procedure could be safely performed in the second trimester. This is not advisable due to the significant risks involved; elective surgeries should ideally be postponed until postpartum [37].

There were other points noted during our review of the answers from ChatGPT-4o mini, including literal translation, controversial answers, and commercial recommendations. ChatGPT-4o mini sometimes makes poor translations from English to Arabic (literal translation), for example, with the words aloe vera and plaque. ChatGPT-4o mini has provided controversial answers on different occasions. For example, it emphasized in one answer the importance of performing dental treatment during the second trimester of pregnancy, considering it the safest route, while in another answer it encourages performing dental treatments in the third trimester, specifically, the seventh month. It also has recommended commercial products in more than one inquiry. We could ask what parameters led ChatGPT to recommend these products and not others.

This might explain the need for a professional who can help patients make wise decisions. In fact, most of the time, ChatGPT-4o mini recommends that patients consult with a dental professional and should not take ChatGPT’s answers as definitive, which is considered to be good practice. Nevertheless, the public might not take this advice and use such information without consulting a dental professional due to the free cost of the service and easy access to such information and responses. As ChatGPT-4o mini showed promise in usability, its role in direct patient interactions should be approached with caution. The responses were not always consistent or fully accurate as detailed above, and the system lacks the ability to tailor advice to individual health contexts, as it gives answers most of the time without asking for further investigation of the precise care. These limitations highlight the importance of using such tools as supportive resources, rather than as standalone sources of medical guidance.

The information was evaluated by calibrated senior dental students (near graduation) under the supervision of a dental public health professor. As the questions were mostly general and not highly specialized, they fell within the knowledge scope of general dentists. However, to improve assessment reliability, involving licensed dentists or oral medicine specialists is recommended for future studies.

### Part 2: usability of ChatGPT

Regarding the usability of ChatGPT, our results indicated that 42.0% (126/300) of participants were aware of ChatGPT, while only 8.3% (25/300) had used it to obtain dental information during pregnancy. This seems to be different from one country to another and from one population to another. There were several recent studies in Saudi Arabia [38, 39], United Arab Emirates [40], and Jordan [41] that found different percentages using ChatGPT. However, it was noticed that there is a gradual increase in the percentage of familiarity and awareness of ChatGPT from 2023 (23.8%) [41] and 2024 (91.0–91.2%) [38, 40] to 2025 (90.1–97.9%) [39]. The studies also indicated that a major percentage of respondents had previously used ChatGPT, ranging from 75.1 to 85.0% [38, 40] in recent years. There is a scarcity of public use of ChatGPT in literature. Nevertheless, our results are similar to a previous study [41] that indicated that awareness of ChatGPT increases with higher levels of education. It seems that a large segment of the Saudi population, especially pregnant women are not using ChatGPT, in comparison to healthcare student populations in other Saudi studies [38, 39]. This might change in the recent fire-hot global AI race. The reason for this might be because Arabic users think that such tools are not suitable for Arabic or because the public did not embrace such changes in technology. This actually was reflected the insignificant difference in usability when compared by several demographic variables in our results. It is worth mentioning that Saudi Arabia is one of the countries that is raising AI investments and initiatives on a global scale [42], which can change the usability of AI chatbots in the near future.

Almost all attitude components regarding ChatGPT usage during pregnancy for oral health were rated positively above the midpoint. The highest one was for ChatGPT competency, and the lowest was regarding the ability of ChatGPT to replace human interactions. None of the attitude components were significantly different against nationality, marital status, pregnancy status, or region. This was similar to a study that assessed Saudi public perceptions and attitudes about AI in healthcare [43]. The overall attitude was positive, with the highest attitude being that most participants were aware of AI, while the lowest attitude was the belief that AI may replace healthcare professionals [43].

However, several attitude components were notably lower among participants with higher education. This may suggest that individuals with advanced education are more likely to be familiar with AI, either due to the nature of their profession or the growing demand for AI-related services. It should be noticed that one of the attitude components was barely significant (*p* =.049). Additionally, their greater awareness of technological limitations and challenges in the AI industry could explain their lower levels of trust in ChatGPT. Nevertheless, some studies have different points of view, as one study found that people with higher education in the United Kingdom had more reliance on AI power recommendations [44]. In fact, this difference might result from measuring different populations and cultures. More studies are needed to verify our findings in contrast to the worldwide body of literature.

This study is considered the first in Saudi Arabia to assess the quality and usability of ChatGPT, as well as attitudes, regarding oral health in the Arabic language. Additionally, the study utilized the latest available ChatGPT models available to the public at the time of the study (ChatGPT-4o mini). However, there are some challenges due to the study’s use of a self-reported questionnaire to assess the information quality, usability and attitude. Despite having participants from different regions in Saudi Arabia, the sampling method imposes difficulties for generalizability of the usability and attitude results, and limits in generalizability to all Arabic-speaking nations. A multicenter study involving cross-cultural Arabic populations with a proportionate sample is recommended for future research to yield more generalizable findings. It is also recommended to recruit participants from multicentered hospital waiting areas, rather than relying solely on social media invitations, to minimize selection bias. Using social media platforms to recruit participants makes it impractical to track the response rate.

Regarding usability and attitude, future studies can investigate cross-cultural differences in different nations, the difference of usability and attitude among different AI chatbot platforms and investigate the general population regarding use of ChatGPT or similar AI chatbots for oral health in general. Regarding the quality of ChatGPT information, future researchers can compare ChatGPT with other AI chatbots growing in popularity, such as Gemini, Anthropic AI, and DeepSeek platforms, to evaluate their quality. Also, different available versions of these platforms should be investigated, as that might bring different results. While we were writing this paper, OpenAI launched new versions of Search and Deep Search modes that are quite different and worth further investigation. It is particularly important to declare the advancement and continuous improvement in AI chatbot platforms are moving at incredible speed that makes the research much harder to validate, especially when new models can evolve within a very short time. Additionally, future studies can investigate the improvement in these chatbots and their ability to compete with consultants and professional answers without supervision.

## Conclusions

ChatGPT-4o mini demonstrated potential in delivering generally acceptable responses to oral health inquiries from Arabic-speaking pregnant women. However, some of its information needed to be revised and supervised by a dental professional. ChatGPT should not replace professional dental advice, especially in vulnerable populations such as pregnant women. The use of ChatGPT for oral health during pregnancy is considerably low in Saudi Arabia. There are positive attitudes regarding using ChatGPT, but some of these attitudes were lower among educated people. Future studies are needed when ChatGPT becomes more familiar with the targeted and other populations. The AI and chatbot area is a rich field of exploration as it is newly emerging with many applications and the potential to improve oral healthcare.

## Supplementary Information

Supplementary Material 1.

Supplementary Material 2.

## Data Availability

The supplemental file for this paper S1 contains all the data produced or analyzed during this investigation.

## Abbreviations

AI: Artificial intelligence
LLM: Large language model
ML: Machine learning
NLP: Natural language processing
