# Supplementary material for: Can ChatGPT be trusted? Evaluating AI responses to oral health questions among pregnant Arabic-speaking women
Source: BMC Oral Health. 2025 Oct 10;25:1597. doi: 10.1186/s12903-025-06909-z (PMC12513007; doi:10.1186/s12903-025-06909-z)
Supplement: Supplementary file 1 — Supplementary Material 1. [file 12903_2025_6909_MOESM1_ESM.pdf]

SN	Age	Nationalit	Region	Marital	Pregnancy	Children	Education
1	25	1	1	1	2	2	3
2	26	1	2	1	1	0	3
3	21	2	3	1	1	2	2
4	28	2	4	1	2	1	3
5	24	1	1	1	1	1	3
6	35	1	2	1	2	6	3
7	30	1	1	1	2	2	4
8	23	2	1	1	2	2	2
9	29	1	2	1	1	0	3
10	30	2	1	1	2	2	3
11	29	1	1	1	2	2	3
12	24	1	1	1	2	1	3
13	33	1	1	1	2	2	3
14	32	1	1	1	2	2	3
15	41	1	1	1	2	2	3
16	45	1	1	1	2	3	3
17	43	1	1	1	2	4	3
18	30	1	1	1	2	2	3
19	27	1	1	1	2	1	3
20	30	1	1	1	2	4	2
21	30	1	1	1	1	2	3
22	35	1	1	1	2	2	3
23	31	1	1	1	1	0	3
24	29	1	1	1	2	1	3
25	30	1	1	1	2	3	3
26	36	1	1	1	1	2	3
27	35	2	1	1	1	2	4
28	23	1	4	1	1	0	3
29	35	1	4	1	2	3	3
30	34	1	1	1	2	2	4
31	24	1	1	1	2	1	4
32	28	2	1	1	2	3	1
33	43	1	4	1	2	4	3
34	33	1	1	1	2	1	3
35	37	2	4	1	1	3	1
36	30	1	1	1	2	1	3
37	24	1	1	1	2	1	3
38	25	1	4	1	1	0	3
39	25	1	2	1	2	3	3
40	25	1	1	1	2	1	3
41	26	1	1	1	2	1	3
42	27	1	1	1	2	2	3
43	23	1	1	1	2	1	3
44	31	1	1	1	1	2	3
45	28	1	1	1	1	4	2
46	38	1	1	1	2	3	3

47	36	1	1	1	2	6	2
48	20	1	4	1	1	0	2
49	40	2	1	2	2	1	2
50	21	1	2	1	1	0	3
51	28	1	1	1	2	3	3
52	40	1	1	1	2	1	3
53	37	1	1	1	1	2	4
54	44	1	1	1	2	5	3
55	45	1	1	1	1	5	3
56	33	1	4	1	2	3	3
57	28	1	1	1	1	0	3
58	32	1	1	1	2	3	4
59	30	1	1	1	2	2	3
60	35	1	1	1	1	2	3
61	27	1	1	1	1	2	3
62	30	1	1	1	2	2	3
63	29	1	1	2	2	2	2
64	30	1	1	1	2	2	3
65	31	1	1	1	2	6	2
66	29	1	1	1	2	3	3
67	19	1	1	1	1	0	2
68	26	1	1	1	1	0	3
69	30	1	1	1	1	1	3
70	30	1	4	1	1	2	3
71	22	1	3	1	1	0	3
72	23	1	3	1	2	1	3
73	36	1	2	1	2	4	3
74	26	1	1	1	1	1	3
75	25	1	4	1	2	1	3
76	31	1	2	1	1	3	2
77	23	1	1	1	2	1	2
78	23	1	3	1	2	2	3
79	26	1	3	1	1	0	3
80	30	1	1	1	1	1	3
81	28	1	1	1	2	4	2
82	27	2	2	1	1	4	1
83	40	1	1	1	1	2	2
84	25	1	1	1	1	0	3
85	21	1	3	1	2	1	3
86	30	1	1	1	1	2	3
87	23	2	2	1	1	1	1
88	26	1	2	1	1	1	3
89	24	1	1	1	2	0	3
90	30	1	2	1	2	2	4
91	25	1	1	1	1	0	3
92	24	1	1	1	2	1	3
93	35	1	1	1	2	4	4
94	35	1	3	1	2	3	4
95	35	1	1	1	1	3	4

96	30	2	1	1	1	0	2
97	25	1	3	1	2	1	3
98	29	1	1	1	1	2	3
99	38	2	1	1	1	2	2
100	29	1	2	1	1	0	3
101	25	1	1	1	1	0	3
102	22	2	5	1	1	0	3
103	27	1	1	1	1	0	3
104	21	1	1	1	2	1	2
105	23	1	1	1	1	1	3
106	22	1	1	1	2	1	3
107	24	1	2	1	1	1	3
108	24	1	2	1	1	0	3
109	24	1	1	1	2	1	3
110	40	1	1	1	2	3	2
111	25	1	1	1	2	1	3
112	27	1	1	1	1	0	3
113	22	1	2	1	2	1	3
114	36	2	1	1	2	3	3
115	36	1	1	1	2	5	2
116	44	1	1	1	2	5	2
117	20	1	1	1	1	0	3
118	34	1	1	1	1	2	2
119	27	1	2	1	2	1	3
120	40	2	1	1	2	1	2
121	25	2	1	1	2	1	3
122	30	1	1	1	1	0	2
123	21	1	4	1	2	1	2
124	27	1	1	1	1	0	3
125	27	1	1	1	1	2	4
126	21	1	5	1	1	0	3
127	22	2	2	1	1	1	2
128	30	1	1	1	1	0	3
129	24	1	4	1	2	1	3
130	32	1	3	1	1	2	3
131	32	1	2	1	1	0	3
132	23	1	3	1	2	1	3
133	25	1	1	1	2	2	2
134	27	1	1	1	1	0	3
135	22	1	5	1	1	0	3
136	29	1	2	1	1	2	3
137	23	1	3	1	1	2	2
138	20	2	3	1	1	0	3
139	27	1	1	1	1	0	3
140	27	1	3	1	2	1	4
141	24	1	2	1	1	1	3
142	36	1	2	1	2	1	2
143	27	1	1	1	1	0	3
144	22	1	4	1	2	2	3

145	21	1	4	1	1	1	2
146	24	1	3	1	2	2	3
147	35	1	2	1	1	1	3
148	23	2	3	1	1	2	1
149	27	1	5	1	1	3	3
150	29	1	2	1	1	0	3
151	24	1	2	1	2	1	3
152	30	1	3	1	2	3	3
153	26	1	1	1	1	1	3
154	24	1	4	1	1	0	3
155	28	1	1	1	2	2	3
156	30	1	1	1	1	0	4
157	22	1	1	1	2	1	3
158	25	1	1	1	1	0	3
159	33	1	1	1	2	3	3
160	35	1	1	1	1	3	3
162	35	1	1	1	1	3	3
163	23	2	1	1	1	0	2
164	38	1	1	1	2	4	3
165	39	1	2	1	1	5	2
166	40	1	1	1	2	5	3
167	34	1	1	1	2	4	3
168	34	1	1	1	1	3	3
169	25	1	1	1	2	0	3
170	36	1	1	1	2	3	1
171	31	1	5	1	1	2	2
172	32	1	5	1	2	1	3
173	30	1	2	1	2	1	3
174	32	1	1	1	2	2	3
175	33	1	1	1	2	3	4
176	35	1	1	1	2	8	3
178	46	1	1	2	2	4	3
179	40	1	1	1	2	7	3
180	20	1	2	1	2	1	3
181	34	1	1	1	1	2	3
182	26	1	1	1	1	0	3
183	23	2	4	1	2	1	3
184	21	2	2	1	1	1	3
185	39	1	2	1	1	4	2
186	27	1	4	1	1	1	2
187	26	1	2	1	1	0	3
188	27	1	4	1	1	2	3
189	33	1	1	1	1	2	3
190	33	1	1	1	1	2	3
191	24	1	4	1	1	1	2
192	23	1	2	1	1	0	3
193	40	1	1	1	2	5	3
194	28	1	1	1	2	3	2
195	43	1	4	1	2	6	3

196	28	1	1	1	1	0	3
197	46	1	1	1	2	3	3
198	23	1	2	1	1	0	2
199	32	1	1	1	2	4	3
200	22	1	1	1	1	0	3
201	45	2	4	1	1	4	3
202	22	1	3	1	2	1	3
203	21	1	1	1	2	1	3
204	37	1	5	1	2	5	3
205	36	1	1	1	2	4	3
206	34	1	2	1	1	1	3
207	33	1	2	1	1	0	3
208	25	1	1	1	1	1	3
209	28	1	3	1	1	1	2
210	26	1	1	1	1	0	3
211	26	2	2	1	2	1	3
212	27	1	1	1	2	1	3
213	26	1	1	1	1	0	3
214	23	2	3	1	2	1	3
215	28	1	2	1	1	1	3
216	32	1	5	1	1	1	3
217	37	1	5	1	2	5	3
218	20	1	1	1	2	1	3
219	24	1	1	1	2	2	3
220	27	1	3	1	2	1	3
221	24	1	1	1	2	1	3
222	30	1	2	1	2	1	3
223	22	1	2	1	1	0	3
224	30	1	3	1	1	0	3
225	23	1	1	1	1	0	3
226	30	1	3	1	2	3	3
227	30	1	3	1	2	3	3
228	23	2	4	1	1	0	3
229	30	1	2	1	1	3	3
230	34	1	3	1	2	4	3
231	24	1	2	1	1	0	3
232	34	1	5	1	2	4	3
233	43	1	2	1	1	5	3
234	26	2	4	1	1	0	3
235	35	1	1	1	2	3	3
236	40	1	1	1	1	0	3
237	22	2	4	1	1	1	2
238	30	1	2	1	1	4	2
239	42	2	1	1	2	1	4
240	21	1	4	1	1	0	2
241	33	2	1	1	1	5	3
242	34	1	1	1	1	5	3
243	22	2	5	1	2	1	2
244	21	2	2	1	1	0	1

245	27	1	5	1	1	1	4
246	24	1	2	1	1	0	3
247	22	1	3	1	1	0	3
248	21	1	1	1	1	0	3
249	33	1	2	1	1	0	3
250	23	1	4	1	1	0	3
251	38	1	1	1	2	2	4
252	27	1	1	1	2	1	3
253	32	1	5	1	1	2	4
254	40	2	4	1	2	3	1
255	29	1	1	1	2	1	3
256	31	1	2	1	1	3	3
257	24	1	3	1	1	0	3
258	30	1	3	1	1	1	3
259	22	2	4	1	1	0	2
260	40	1	2	1	2	4	2
261	29	1	1	1	1	1	3
262	25	1	1	1	1	0	2
263	36	1	1	1	2	4	3
264	26	2	2	1	1	0	3
265	26	1	1	1	1	0	4
266	21	1	4	1	2	1	3
267	33	1	2	1	1	2	3
268	20	1	2	1	2	1	2
269	25	1	1	1	1	1	2
270	25	1	5	1	1	0	3
271	27	1	1	1	1	2	3
272	24	1	2	1	1	0	3
273	22	1	3	1	1	1	2
274	26	1	2	1	1	0	3
275	28	1	4	1	2	4	3
276	25	1	5	1	1	0	3
277	33	1	3	1	2	3	3
278	40	1	1	1	2	6	3
279	25	1	3	1	1	0	3
280	38	1	1	1	2	3	3
281	26	1	1	1	1	0	3
282	35	1	1	1	2	2	3
283	24	1	1	1	1	0	2
284	23	2	1	1	1	2	2
285	31	1	2	1	1	0	3
286	24	1	2	1	1	0	3
287	27	2	2	1	1	0	2
288	43	1	1	1	2	3	2
289	22	1	1	1	1	0	3
290	42	1	4	1	2	4	2
291	23	1	4	1	1	0	3
292	25	1	3	1	1	0	4
293	22	1	1	1	1	0	3

294	29	1	1	1	1	0	2
295	24	1	2	1	2	3	3
296	24	1	2	1	1	0	3
297	27	2	1	1	2	1	3
298	24	2	1	1	1	0	3
299	22	2	3	1	1	1	2
300	32	1	2	1	1	1	4
301	25	1	4	1	1	0	3
302	28	1	1	1	2	1	3

Medical_d	Dentist	Family_fri	Social_me	internet	TV	Books	ChatGPT	Others
2	1	1	1	1	2	2	2	2
2	1	1	1	1	1	1	2	1
2	1	1	1	1	2	1	2	2
2	1	2	2	2	2	2	2	2
1	1	1	1	1	2	2	2	2
2	1	2	1	1	1	2	2	2
2	1	1	2	1	2	1	2	1
2	1	1	1	1	2	2	1	1
2	1	2	1	1	2	2	2	1
2	1	1	1	1	2	1	2	2
1	1	2	2	1	2	2	1	1
2	1	2	1	1	2	2	1	2
1	1	1	1	1	1	2	2	1
1	1	1	1	1	2	2	1	2
2	1	1	1	1	1	2	2	2
2	1	2	1	2	1	1	2	2
1	1	1	2	2	2	2	2	2
1	1	2	1	1	2	2	2	2
2	1	1	1	1	2	2	2	2
2	1	1	1	1	2	1	1	1
1	1	1	2	1	2	2	2	2
2	1	1	2	1	2	2	1	2
2	1	2	2	2	2	2	2	2
2	1	2	2	1	2	2	2	2
2	1	2	1	1	2	2	2	1
2	1	2	1	1	1	2	2	2
2	1	1	1	1	2	1	2	2
2	1	1	1	1	2	2	2	2
2	2	2	2	1	2	2	2	2
2	1	2	1	1	2	2	2	1
1	1	1	1	1	2	2	1	2
1	1	2	1	2	2	2	2	1
1	1	1	1	1	2	2	2	1
2	1	1	1	2	2	2	1	2
2	1	1	1	1	2	1	1	1
2	1	1	1	1	1	1	2	1
2	1	1	1	1	1	1	1	1
2	1	2	2	2	2	2	2	2
2	2	2	1	1	2	1	1	2
1	1	2	1	1	2	2	2	2
2	1	1	2	1	2	1	2	2
1	1	1	2	2	2	2	2	2
1	1	1	2	1	2	2	2	2
2	1	2	1	2	1	2	1	1
2	1	1	1	1	1	2	2	2
2	1	2	1	1	2	2	2	2

1	1	2	1	1	1	1	1	1
2	1	2	1	1	2	2	2	1
2	1	2	2	1	2	2	2	1
2	1	2	1	1	1	2	2	1
1	1	1	1	1	1	1	1	1
2	1	2	2	1	2	2	2	2
1	1	2	2	2	1	2	2	2
2	1	1	1	1	2	2	1	2
1	1	2	2	1	2	2	1	1
1	1	2	1	1	2	2	2	2
2	1	1	1	1	2	2	2	1
2	1	1	1	1	2	2	2	2
2	1	2	1	1	2	2	2	2
2	2	1	1	1	1	2	2	2
1	1	2	1	1	2	1	2	2
2	1	1	1	1	2	2	2	2
1	2	1	1	2	2	2	1	2
2	1	1	1	1	2	2	2	2
1	1	1	1	1	1	1	2	2
2	1	2	1	1	1	2	2	2
1	1	1	1	1	1	1	2	2
2	1	1	1	1	2	2	2	2
1	1	1	2	1	2	2	2	2
2	1	2	1	2	2	2	2	2
2	1	1	1	1	2	2	2	2
1	1	1	2	2	2	2	2	1
2	1	2	1	1	1	2	2	2
2	1	1	1	1	2	2	2	2
2	1	1	1	1	2	2	2	2
1	1	2	2	2	2	2	2	2
2	1	1	2	1	2	2	2	2
2	1	1	1	2	2	1	2	2
2	1	1	1	1	2	2	2	2
1	1	1	1	2	2	2	2	2
2	1	1	1	2	2	2	2	2
2	1	1	1	1	2	2	2	2
2	1	2	2	1	2	2	2	1
2	1	2	1	2	2	2	2	2
1	1	2	1	1	1	1	2	2
1	1	1	1	2	2	2	2	2
2	1	1	1	1	2	2	1	1
1	1	1	1	1	1	1	2	2
2	1	1	1	1	2	2	2	2
2	1	1	1	1	1	1	1	2
2	1	1	1	1	2	2	2	2
2	1	1	1	1	1	1	1	2
1	1	2	1	1	2	2	2	2
2	1	1	1	1	1	1	2	2
1	1	1	1	2	2	2	2	2

2	1	1	1	1	2	2	2	1
2	1	1	1	1	2	2	2	2
2	1	2	1	1	1	1	2	2
1	1	1	1	1	1	1	1	2
1	1	1	2	2	2	2	2	1
1	1	1	1	2	2	2	2	2
2	1	2	2	2	2	2	2	2
1	1	1	1	1	1	1	2	2
1	1	2	1	1	2	2	2	2
2	1	1	1	1	2	1	2	1
1	1	1	1	1	2	2	2	1
2	1	2	1	1	2	2	2	1
1	1	1	1	1	2	2	2	1
2	1	2	1	1	2	2	1	2
2	1	2	1	1	2	2	2	2
2	1	2	1	2	2	2	2	2
2	2	1	1	1	2	2	2	2
2	1	1	1	1	2	2	2	2
2	1	1	1	1	2	2	2	1
2	1	2	1	1	2	1	2	1
2	1	1	1	1	1	1	1	1
2	1	1	1	1	2	1	2	2
1	2	1	1	1	2	2	2	1
2	1	2	1	1	1	2	2	2
2	1	1	2	2	2	2	2	2
2	1	2	1	1	1	1	2	2
2	1	1	1	1	2	2	2	2
2	1	1	1	2	2	1	2	1
1	1	1	1	1	1	1	2	2
2	1	1	1	1	2	2	2	2
1	1	1	2	1	2	2	2	2
2	1	1	1	1	2	2	2	2
2	1	2	1	1	2	2	2	2
2	1	1	1	1	1	2	2	1
1	1	2	1	2	2	1	2	2
2	1	2	1	1	2	2	2	2
2	1	1	1	1	2	1	2	2
2	1	1	1	1	2	2	2	1
2	1	1	1	1	1	1	1	2
1	1	1	1	1	2	2	2	2
1	1	2	1	2	2	2	2	2
2	1	1	1	1	1	1	2	2
1	2	1	1	2	2	1	1	2
2	1	1	1	2	2	2	2	1
2	1	2	1	1	2	2	2	1
2	1	2	1	1	2	2	2	2
2	1	2	1	1	1	2	2	2
2	1	1	1	1	2	2	2	2
2	1	1	1	1	2	1	2	2

2	1	2	1	1	2	2	2	1
2	1	2	1	2	1	1	2	1
2	1	1	2	1	2	2	2	2
2	1	2	2	2	2	2	2	2
1	1	1	1	1	2	2	2	1
2	1	2	2	2	2	1	2	2
2	1	2	1	1	2	1	2	1
1	1	2	2	1	1	1	2	1
2	1	1	1	1	2	2	2	2
2	1	2	1	1	2	2	2	2
1	1	1	1	2	2	1	2	2
1	1	1	2	2	2	2	2	2
2	1	2	2	1	2	2	2	2
1	1	1	1	2	2	2	2	2
2	1	1	1	1	1	2	2	2
2	1	2	1	2	2	2	2	2
2	1	2	2	2	2	2	2	2
2	1	2	1	1	1	1	2	1
2	1	1	2	1	2	1	2	2
2	1	1	1	1	2	2	2	2
2	1	1	1	1	2	1	1	2
2	1	1	2	1	2	2	1	1
1	1	1	1	1	1	1	2	2
2	1	2	1	1	2	2	2	2
2	1	1	1	1	2	2	2	2
1	1	1	1	1	2	2	2	1
2	1	1	1	1	2	2	2	1
1	1	2	1	2	2	2	2	2
1	1	1	1	1	2	2	2	1
2	2	1	1	1	2	2	2	2
2	1	1	1	1	2	2	2	2
2	1	1	1	1	1	1	2	1
2	1	1	1	1	1	1	2	2
2	1	2	1	1	2	1	2	2
1	1	1	1	1	2	1	1	2
2	1	1	1	1	2	2	2	2
2	1	1	1	2	2	2	2	2
1	1	1	1	1	2	2	2	2
2	1	1	1	1	1	1	2	2
2	1	1	1	1	2	2	2	2
2	1	1	1	1	2	2	2	2
2	1	1	1	2	1	1	2	1
2	1	1	1	1	2	2	2	2
2	1	1	1	1	2	2	2	2
1	1	1	1	1	1	1	2	2
2	1	2	1	1	2	1	2	2
1	1	2	2	1	2	1	2	2
1	1	2	1	1	2	2	2	2
2	1	1	2	1	2	1	2	2

1	1	2	2	1	2	2	2	2
1	1	1	1	1	1	1	1	1
1	1	1	2	2	2	2	2	2
1	1	1	1	1	2	2	2	1
2	1	2	1	1	2	2	2	1
1	1	1	1	1	1	1	2	2
2	1	2	1	1	2	2	1	1
2	1	1	2	1	2	1	1	1
1	1	2	1	1	1	2	2	2
1	1	1	1	1	1	2	2	2
2	1	2	1	2	2	2	1	2
1	1	2	1	1	2	2	2	2
2	1	1	1	2	2	2	2	2
2	1	1	2	1	2	2	2	2
2	1	1	1	1	2	2	1	2
2	1	1	1	1	2	2	2	1
1	1	2	1	1	2	2	2	2
2	1	1	1	1	2	2	1	2
1	1	2	1	1	2	2	2	1
1	1	1	2	2	2	2	2	2
2	1	2	2	1	2	1	2	2
1	1	1	1	1	1	1	1	1
1	1	1	1	1	2	2	2	2
2	1	1	1	1	1	1	2	2
1	1	1	1	2	2	2	2	2
1	1	2	2	2	2	1	1	2
2	1	1	1	1	2	2	2	2
1	1	2	2	2	2	2	2	2
2	1	2	1	1	2	2	2	2
2	1	1	2	2	2	2	2	2
2	1	2	2	1	2	1	2	2
2	1	2	2	1	2	1	2	2
1	1	2	1	1	2	2	2	1
2	1	2	1	1	2	1	1	1
1	1	1	2	1	1	2	2	2
2	1	2	1	1	1	2	2	2
2	1	1	1	1	2	2	2	2
2	1	1	1	1	2	2	2	2
1	1	1	1	1	1	1	2	2
2	1	1	1	1	2	2	2	2
1	1	1	1	1	2	2	2	2
1	1	1	1	1	1	2	2	2
1	1	1	1	1	1	1	2	1
2	1	2	1	1	1	2	2	2
1	1	2	2	1	2	1	2	1
2	1	2	1	1	2	1	2	2
2	1	1	2	1	1	1	2	2
2	1	1	1	1	2	2	2	1
2	1	2	2	2	2	2	2	2

2	1	1	2	2	2	1	2	1
1	1	2	1	1	2	2	2	1
2	1	2	2	1	2	2	1	2
2	1	2	1	1	2	2	2	2
1	1	1	2	2	2	2	2	2
2	1	1	1	1	2	2	2	2
1	1	2	1	1	1	2	2	2
1	1	1	1	2	2	2	2	2
2	1	1	1	1	2	2	2	2
1	1	1	1	2	2	2	2	2
2	1	1	1	1	2	2	2	2
1	1	1	2	2	2	2	2	1
2	1	1	1	1	1	2	2	1
1	1	1	1	1	1	2	2	2
1	1	1	2	2	2	2	2	2
1	1	1	1	1	1	2	2	1
2	1	2	1	1	2	2	2	2
2	1	2	1	1	2	2	2	2
1	1	1	2	2	2	2	1	2
1	1	1	1	1	1	2	1	1
1	1	2	1	1	2	1	1	1
2	1	2	2	1	2	2	2	2
2	1	1	2	2	2	2	2	2
2	1	1	1	1	1	1	2	2
2	1	1	1	1	2	1	2	2
2	1	2	1	1	2	2	2	2
2	1	2	1	1	2	2	2	1
2	1	2	1	1	2	2	1	2
2	1	1	1	2	2	2	2	2
2	1	1	1	2	2	2	2	2
2	1	1	1	1	2	2	2	1
2	1	2	2	2	2	2	2	2
2	1	1	1	2	1	1	2	1
2	1	1	1	2	2	2	2	2
2	1	1	1	1	2	2	2	2
2	1	1	1	2	2	2	2	2
2	1	2	2	2	2	2	2	2
2	1	2	2	2	2	1	2	2
2	1	1	2	2	2	2	2	2
2	1	2	1	1	2	2	2	1
2	1	2	2	1	2	1	2	2
2	1	2	1	2	2	2	2	2
1	2	1	1	1	2	2	2	1
2	1	2	1	1	2	2	2	2
1	1	2	1	1	2	2	2	1
2	1	1	1	1	2	1	1	2
1	1	1	1	1	1	1	1	1
2	2	1	1	1	2	2	2	2
2	1	2	2	2	2	1	2	2

2	1	2	2	2	2	2	2	2
1	1	2	2	2	2	2	2	1
2	2	1	1	1	2	2	2	2
2	1	2	1	1	2	2	1	2
1	1	1	1	1	1	2	2	2
2	1	2	2	2	2	2	1	2
2	1	2	1	1	2	2	2	2
2	1	2	2	1	2	2	2	2
2	1	1	1	1	2	2	2	2

A1	A2	A3	A4	A5	B1	B2	B3	B4
2	2	2	2	2	3	2	2	2
2	2	2	2	2	3	3	3	3
1	2	2	2	2	3	3	2	2
1	1	1	2	2	3	3	3	2
2	2	2	2	2	3	3	3	3
2	2	2	2	2	1	1	1	1
2	2	2	2	2	3	3	3	3
1	1	1	1	1	3	3	3	3
1	1	1	2	2	3	3	3	3
1	1	2	2	2	3	3	2	3
1	1	2	2	2	3	3	4	4
1	1	1	1	1	3	3	4	3
2	2	2	2	2	1	1	1	1
1	1	1	1	1	4	4	4	4
2	2	2	2	2	2	2	3	2
2	2	2	2	2	3	2	3	3
1	1	2	2	2	3	3	3	3
2	2	2	2	2	3	3	3	3
1	2	2	2	2	2	2	2	2
1	1	1	1	1	3	3	3	3
1	1	1	2	2	3	3	3	3
2	2	2	2	2	3	3	3	3
1	1	2	2	2	3	3	3	3
2	2	2	2	2	3	3	3	3
2	2	2	2	2	3	3	3	3
2	2	2	2	2	3	3	3	3
2	2	2	2	2	3	3	3	3
2	2	2	2	2	3	3	3	3
2	2	2	2	2	3	3	3	3
2	2	2	2	2	2	2	2	2
1	1	1	1	1	1	1	1	1
2	2	2	2	2	2	1	2	1
2	2	2	2	2	2	2	2	2
2	2	2	2	2	3	2	3	3
2	2	2	2	2	3	3	3	3
2	2	2	2	2	3	3	3	3
1	1	1	2	2	1	1	1	1
2	2	2	2	2	3	3	3	3
1	1	1	1	1	2	2	2	2
1	1	2	2	2	3	2	3	3
1	1	2	2	2	1	1	1	1
2	2	2	2	2	4	4	4	4
1	1	2	2	2	2	2	2	2
2	2	2	2	2	3	3	3	3
1	1	1	2	2	4	3	4	4
2	2	2	2	2	3	3	3	3

1	1	1	1	1	4	4	4	4
1	1	1	2	1	3	3	3	3
2	2	2	2	2	4	4	4	4
1	1	1	1	1	3	3	3	3
1	1	1	2	2	4	4	3	3
1	1	2	2	2	3	2	3	2
1	1	2	2	2	2	2	2	2
1	1	2	1	2	2	1	2	1
1	1	1	2	2	4	3	3	3
1	1	2	2	2	3	3	3	3
2	2	2	2	2	3	3	3	3
1	2	2	2	2	3	3	3	3
2	2	2	2	2	4	4	4	4
2	2	2	2	2	2	3	2	2
1	1	2	2	2	2	2	3	1
2	2	2	2	2	3	3	3	3
2	1	2	2	1	1	4	1	4
1	1	2	2	2	3	3	3	3
2	2	2	2	2	2	1	3	2
2	2	2	2	2	3	3	3	3
2	2	2	2	2	2	2	2	2
2	2	2	2	2	3	3	3	3
1	2	2	2	2	3	2	3	2
2	2	2	2	2	2	2	2	2
2	2	2	2	2	1	3	1	3
2	2	2	2	2	3	3	3	3
1	2	2	2	2	2	2	2	2
1	2	2	2	2	2	3	3	3
2	2	2	2	2	3	1	1	3
2	2	2	2	2	3	3	3	3
2	2	2	2	2	2	2	2	2
2	2	2	2	2	2	2	2	2
1	1	2	2	2	1	1	1	1
2	2	2	2	2	3	3	3	3
2	2	2	2	2	3	3	3	1
2	2	2	2	2	3	2	3	2
2	2	2	2	2	3	2	2	2
2	2	2	2	2	3	3	3	3
2	2	2	2	2	3	3	3	3
2	2	2	2	2	1	1	1	1
2	2	2	2	2	3	2	3	2
1	1	2	2	2	3	2	3	3
1	1	1	2	2	1	1	1	1
1	2	2	2	2	3	3	3	3
2	2	2	2	2	3	3	2	1
2	2	2	2	2	3	2	2	2
2	2	2	2	2	3	2	3	3
1	2	2	2	2	3	3	3	3
2	1	2	2	2	3	2	3	3

2	2	2	2	2	2	2	3	1
2	2	2	2	2	3	2	2	2
1	1	2	2	2	4	3	3	3
1	1	2	2	2	3	3	3	3
1	1	2	2	2	3	3	3	3
2	2	2	2	2	3	3	4	3
2	2	2	2	2	3	3	3	3
1	2	2	2	2	3	3	3	3
2	2	2	2	2	3	3	3	3
1	2	2	2	2	3	3	3	3
2	2	2	2	2	3	3	3	3
2	2	2	2	2	3	3	3	3
2	2	2	2	2	3	3	3	3
1	1	1	1	1	1	1	1	1
2	2	2	2	2	3	3	3	3
2	2	2	2	2	3	4	3	3
1	1	2	2	2	2	2	3	2
1	1	2	2	2	3	3	3	2
2	2	2	2	2	3	3	3	3
2	2	2	2	2	3	4	3	3
1	2	2	2	2	3	2	4	3
1	1	2	2	2	4	4	3	4
2	2	2	2	2	3	3	3	3
2	2	2	2	2	3	3	3	3
2	2	2	2	2	3	3	3	3
1	2	2	2	2	3	4	3	2
2	2	2	2	2	3	3	3	3
1	1	2	2	2	2	2	2	2
1	1	2	2	2	3	2	3	1
1	2	2	2	2	2	2	2	2
2	2	2	2	2	3	3	3	3
2	2	2	2	2	3	3	3	3
1	1	1	2	2	3	2	1	1
1	2	2	2	2	2	3	1	2
2	2	2	2	2	1	1	1	1
2	2	2	2	2	3	4	4	4
2	2	2	2	2	3	3	3	3
2	2	2	2	2	3	2	3	2
1	1	2	2	2	2	2	3	2
2	2	2	2	2	3	3	3	3
2	2	2	2	2	2	1	2	4
1	1	1	1	1	3	4	3	3
1	1	1	1	1	2	2	2	2
1	1	2	2	2	3	3	3	3
1	2	2	2	2	1	2	2	3
1	1	2	2	2	3	3	3	3
2	2	2	2	2	3	2	3	2
1	1	2	2	2	3	3	3	3
2	2	2	2	2	3	3	3	3

2	2	2	2	2	3	3	3	3
1	1	1	2	1	3	2	4	1
2	2	2	2	2	4	4	4	4
2	2	2	2	2	3	3	3	3
2	2	2	2	2	3	3	2	2
2	2	2	2	2	3	3	4	2
2	2	2	2	2	3	3	3	3
2	2	2	2	2	2	3	3	3
2	2	2	2	2	3	3	3	3
2	2	2	2	2	3	3	3	3
1	2	2	2	2	2	2	2	2
2	2	2	2	2	3	3	3	3
2	2	2	2	2	1	1	1	1
1	1	2	2	2	3	2	4	3
2	2	2	2	2	3	3	3	3
1	1	2	2	2	3	3	3	2
2	2	2	2	2	2	2	2	2
2	2	2	2	2	3	2	2	2
2	2	2	2	2	2	2	2	2
2	2	2	2	2	1	2	2	2
2	2	2	2	2	1	1	1	1
1	1	1	1	2	3	3	3	3
2	2	2	2	2	3	3	3	3
2	2	2	2	2	3	3	3	3
2	2	2	2	2	2	2	2	2
2	2	2	2	2	3	4	3	3
2	2	2	2	2	1	2	2	3
1	1	2	2	2	2	2	2	2
2	2	2	2	2	3	4	4	4
1	1	2	2	2	3	3	3	1
2	2	2	2	2	4	3	3	3
2	2	2	2	2	3	3	3	3
2	2	2	2	2	3	3	3	3
1	2	2	2	2	3	3	3	3
1	2	2	2	2	3	3	3	3
2	2	2	2	2	3	3	3	3
2	2	2	2	2	2	2	2	2
1	1	2	2	2	3	3	3	2
2	2	2	2	2	2	3	1	2
2	2	2	2	2	3	4	3	4
2	2	2	2	2	3	3	3	3
2	2	2	2	2	3	3	3	3
2	2	2	2	2	2	2	2	2
2	2	2	2	2	2	2	2	2
1	1	1	2	2	3	3	3	3
1	2	2	2	2	2	1	2	1
2	2	2	2	2	2	3	2	3
2	2	2	2	2	3	3	3	3
2	2	2	2	2	3	3	2	2

2	2	2	2	2	3	3	3	3
2	2	2	2	2	3	3	3	3
2	2	2	2	2	2	2	2	2
2	2	2	2	2	3	3	2	2
2	2	2	2	2	3	3	3	3
1	1	1	1	1	3	3	3	3
1	1	1	2	2	2	1	3	2
1	1	2	2	2	3	3	3	3
2	2	2	2	2	1	1	1	1
2	2	2	2	2	3	2	3	2
1	1	1	1	1	3	3	3	3
1	2	2	2	2	3	2	3	2
1	1	2	2	2	2	2	2	2
2	2	2	2	2	3	3	3	3
1	1	2	2	2	3	3	3	3
1	1	2	2	2	3	2	3	3
1	1	2	2	2	2	2	2	2
1	1	2	2	2	3	3	3	3
1	1	2	2	2	3	2	3	2
1	1	2	2	2	3	3	3	3
2	2	2	2	2	3	3	3	3
1	1	1	1	1	1	1	1	1
1	1	2	2	1	3	3	3	3
2	2	2	2	2	4	3	4	4
2	2	2	2	2	3	3	3	2
1	2	2	2	2	3	3	2	3
1	2	2	2	2	3	2	2	2
1	1	2	2	2	3	3	3	2
2	2	2	2	2	3	3	3	3
2	2	2	2	2	4	4	4	4
2	2	2	2	2	3	3	3	3
2	2	2	2	2	3	3	3	3
2	2	2	2	2	3	3	3	3
2	2	2	2	2	3	3	3	3
2	2	2	2	2	2	2	2	2
1	1	2	2	2	1	2	1	2
2	2	2	2	2	4	4	4	4
1	2	2	2	2	3	3	3	3
2	2	2	2	2	1	1	1	1
2	2	2	2	2	3	2	3	1
2	2	2	2	2	4	4	4	4
2	2	2	2	2	3	3	3	3
2	2	2	2	2	3	3	3	3
1	2	2	2	2	2	2	2	2
2	2	2	2	2	4	4	4	4
2	2	2	2	2	3	3	3	3
2	2	2	2	2	4	3	4	4
2	2	2	2	2	1	1	1	1
2	2	2	2	2	4	1	1	1

1	2	2	2	2	1	1	1	1
2	2	2	2	2	3	3	3	2
1	1	1	1	2	3	2	3	2
1	1	2	2	2	2	2	2	3
2	2	2	2	2	1	1	1	1
1	1	2	2	2	3	2	3	2
2	2	2	2	2	3	3	3	3
1	1	2	2	2	3	3	3	2
1	1	1	1	1	3	3	3	3
2	2	2	2	2	3	3	3	3
1	2	2	2	2	3	1	1	1
1	1	1	1	2	4	4	1	2
2	2	2	2	2	4	4	4	4
2	2	2	2	2	3	2	2	2
2	2	2	2	2	3	3	3	2
2	2	2	2	2	3	3	3	3
2	2	2	2	2	3	3	3	3
1	1	2	2	2	3	3	3	2
1	1	1	1	2	3	3	3	3
1	1	1	1	1	3	3	4	3
1	1	1	1	1	1	1	1	1
1	1	2	2	2	2	2	2	2
1	1	2	2	2	2	2	2	2
1	1	2	2	2	2	2	3	2
2	2	2	2	2	3	3	3	3
2	2	2	2	2	2	2	2	2
2	2	2	2	2	3	4	3	3
1	1	1	1	1	3	2	3	2
1	1	1	2	2	4	4	3	2
1	1	2	2	2	2	2	2	2
1	1	2	2	2	4	4	4	3
2	2	2	2	2	3	3	3	3
1	1	2	2	2	4	4	4	4
2	2	2	2	2	2	2	2	2
1	1	2	2	2	3	3	3	3
1	1	2	2	2	3	3	3	3
1	2	2	2	2	3	2	2	2
2	2	2	2	2	2	2	2	2
2	2	2	2	2	3	3	3	2
2	2	2	2	2	3	3	3	3
1	1	2	2	2	2	1	2	2
1	1	1	2	2	3	3	3	3
2	2	2	2	2	3	3	3	3
2	2	2	2	2	3	3	3	3
2	2	2	2	2	1	1	1	1
1	1	1	1	1	3	3	3	3
1	1	1	1	1	4	4	4	4
2	2	2	2	2	1	1	1	1
2	2	2	2	2	1	1	1	1

2	2	2	2	2	3	3	2	3
1	1	1	1	1	1	1	1	1
2	2	2	2	2	3	2	4	2
1	1	1	2	2	4	4	4	4
2	2	2	2	2	3	3	3	3
1	1	1	2	2	3	2	2	2
1	1	1	2	2	3	3	3	3
2	2	2	2	2	3	3	3	3
2	2	2	2	2	2	3	2	3

B5	B6	B7	B8	B9	B10	B11	B12	B13
2	2	3	3	3	2	2	2	2
3	3	3	3	3	3	3	3	3
3	2	2	2	2	3	2	3	2
3	3	3	3	3	3	3	3	1
3	3	3	3	3	3	3	3	3
1	1	1	1	1	1	1	1	1
2	2	3	3	3	3	3	3	3
3	3	3	3	3	3	3	3	3
3	3	3	3	3	3	3	3	2
1	3	3	2	3	2	2	2	1
2	3	3	3	3	3	3	3	3
4	4	4	3	3	3	3	3	2
1	1	1	1	1	1	1	1	1
4	4	4	3	4	4	4	4	4
2	2	2	2	3	2	2	2	2
3	3	3	3	3	3	3	3	3
2	3	3	2	3	3	3	3	3
3	3	3	3	3	3	3	3	3
2	2	2	2	2	2	2	2	2
3	3	3	2	2	2	2	2	2
3	3	3	3	3	3	3	3	3
2	3	3	3	3	3	3	3	3
3	3	2	2	2	2	2	2	2
3	3	3	3	3	3	3	3	2
3	3	3	3	3	3	3	3	3
3	3	3	3	3	3	3	3	3
2	3	3	3	3	3	3	3	3
3	3	3	3	3	3	3	3	3
3	3	3	3	3	3	3	3	2
3	3	2	3	2	2	2	2	2
1	1	1	1	1	1	1	1	1
2	4	4	2	3	3	4	3	3
2	2	2	2	2	2	2	2	2
2	2	3	3	3	2	3	3	3
3	3	3	3	3	3	3	3	3
3	3	3	3	3	3	3	3	3
1	1	1	1	1	1	1	1	1
3	3	3	3	3	3	3	3	3
4	4	1	2	1	2	2	2	2
4	4	3	2	3	3	3	3	1
1	1	1	3	1	1	1	1	1
4	4	4	4	4	4	4	4	4
3	4	2	3	3	2	3	2	2
2	4	4	3	3	3	3	3	2
3	4	4	4	4	2	4	4	1
3	3	3	3	3	3	3	3	3

4	4	4	4	4	4	4	4	4
3	3	3	3	3	3	3	3	3
4	4	4	4	4	4	4	4	4
3	4	3	3	3	3	3	3	3
3	3	3	3	3	3	3	3	3
3	3	3	3	3	2	3	3	2
2	3	2	2	2	1	2	2	2
1	1	1	1	1	1	1	1	1
3	3	3	3	3	3	3	3	1
3	2	3	3	3	3	3	3	2
3	3	3	3	3	3	3	3	3
3	3	2	3	1	3	3	3	3
4	4	4	4	4	4	4	4	4
2	3	3	2	2	2	2	2	3
2	1	3	2	2	2	3	2	3
3	3	3	4	4	4	4	4	3
1	3	1	1	4	2	3	3	1
3	3	3	3	3	3	3	3	3
3	2	1	2	2	1	2	2	2
4	4	4	3	3	4	4	4	2
2	2	2	2	2	2	2	2	2
3	3	3	3	3	3	3	3	3
3	3	2	3	3	2	3	3	2
2	2	2	2	2	2	2	2	2
1	3	3	3	3	3	3	3	3
3	3	3	3	3	3	3	3	3
2	2	2	2	2	2	2	2	2
2	1	4	4	4	4	4	4	4
1	3	3	3	3	3	3	3	1
3	3	1	3	2	3	2	3	2
2	2	2	2	2	2	2	2	2
3	3	2	3	2	2	2	2	1
1	1	1	1	1	1	1	1	1
3	3	3	3	3	3	3	3	3
1	4	3	2	2	2	2	2	1
3	3	3	3	2	3	2	3	2
2	2	2	2	2	2	2	2	2
3	3	3	3	3	3	3	3	3
4	3	3	3	3	3	3	3	3
2	1	1	1	1	1	1	1	1
3	2	2	2	3	2	2	2	2
2	2	3	3	3	3	2	2	2
1	1	1	1	1	1	1	1	1
3	3	3	3	3	3	3	3	3
4	1	1	1	1	1	4	2	3
2	1	1	1	1	1	1	1	1
3	3	3	3	3	3	3	3	2
3	1	3	2	2	2	3	3	1
3	2	3	3	3	3	3	3	2

2	1	4	3	2	1	1	1	1
2	2	3	3	3	3	3	3	3
3	3	3	3	3	3	3	3	3
3	3	3	3	3	3	3	3	3
2	3	3	2	3	2	3	3	1
3	4	4	4	4	4	3	1	1
3	3	3	3	3	3	3	3	3
2	2	3	3	2	2	3	3	1
3	3	3	3	3	3	3	3	2
3	3	3	3	3	3	3	3	3
3	3	3	3	3	3	3	3	3
3	3	3	3	3	3	3	3	3
3	3	3	3	3	3	3	3	3
1	1	1	1	1	1	1	1	1
4	4	3	3	4	4	3	3	4
1	4	3	3	2	3	3	4	3
3	3	3	2	2	2	3	3	2
2	2	3	3	3	3	3	2	1
3	3	3	3	3	3	3	3	3
3	4	4	3	3	3	3	3	3
4	3	4	3	3	4	2	3	3
4	4	4	4	3	4	4	4	4
3	3	3	3	3	3	3	3	3
3	3	3	3	4	4	3	3	3
3	3	3	3	3	3	3	3	3
3	3	4	4	3	3	3	3	3
3	3	3	3	3	3	3	3	3
2	2	2	2	2	2	3	1	1
2	4	4	2	3	1	3	4	1
2	2	2	2	2	2	2	2	2
3	3	4	3	3	3	3	3	3
3	3	3	3	3	3	3	3	3
4	4	4	4	2	3	3	4	2
1	1	1	1	2	2	1	2	1
1	1	1	1	1	1	1	1	1
4	4	4	4	4	4	4	4	4
2	3	3	3	3	3	3	3	2
2	3	3	3	3	2	3	2	3
3	3	3	2	3	3	3	3	3
3	3	3	3	3	3	3	3	3
2	2	2	2	2	2	2	2	2
1	3	3	3	3	3	3	3	3
2	2	2	2	2	2	2	2	2
3	1	4	2	2	2	2	3	1
2	3	3	2	3	2	3	3	3
3	3	3	3	3	3	3	3	3
2	2	3	2	3	3	3	3	3
3	3	3	3	3	3	3	3	3
3	3	3	3	3	3	3	3	3

2	3	3	3	3	3	3	3	3
3	3	2	4	3	3	4	1	1
4	4	4	4	4	4	4	4	4
3	3	3	3	3	3	3	3	3
2	3	3	3	2	2	3	3	3
3	3	3	3	3	3	3	3	3
4	3	4	3	2	4	3	3	3
2	2	3	3	2	2	2	3	2
3	3	3	3	3	3	3	4	2
3	3	3	3	3	3	3	3	3
2	2	2	2	2	2	2	2	2
3	3	3	3	3	3	3	3	3
1	1	1	1	1	1	1	1	1
3	3	3	2	4	2	3	3	2
3	3	3	3	3	3	3	3	3
3	2	3	3	3	3	3	3	3
2	2	2	2	2	2	2	2	2
2	2	2	2	2	2	2	2	2
2	2	2	2	2	2	2	2	2
2	2	1	1	1	2	1	1	1
1	1	1	1	1	1	1	1	1
3	3	3	3	3	3	3	3	3
3	3	3	3	3	3	3	3	3
3	3	3	3	3	3	3	3	3
2	2	2	2	2	2	2	2	2
3	3	3	3	3	3	3	3	3
2	2	2	2	2	2	2	2	2
3	2	2	2	2	2	2	2	2
4	4	4	4	4	4	4	4	4
1	1	1	1	1	1	2	1	1
3	4	4	3	3	2	3	4	3
3	3	3	3	3	3	3	3	3
3	3	3	3	3	3	3	3	3
3	3	2	3	3	3	3	3	2
2	2	2	3	2	2	3	3	3
3	3	3	3	3	3	3	3	3
2	2	2	2	2	2	2	2	2
2	4	2	3	2	2	2	3	1
2	2	3	3	3	3	3	2	2
3	3	3	3	3	4	4	3	4
3	3	3	2	3	3	3	3	3
2	3	3	3	3	3	3	3	3
2	3	3	2	2	2	2	3	3
2	3	3	2	2	2	2	3	3
3	2	3	2	2	1	3	3	1
3	1	2	2	2	2	1	2	1
2	1	3	3	3	2	2	3	3
3	3	3	3	3	3	3	3	3
3	3	3	3	2	3	3	3	2

3	3	3	3	3	3	3	3	3
3	3	3	3	3	3	3	3	3
2	2	2	2	2	2	2	2	2
3	2	3	2	2	3	3	2	2
3	3	4	3	3	3	3	3	3
2	3	3	3	3	3	3	3	3
2	1	2	1	1	1	1	1	1
2	4	4	3	4	3	4	4	4
1	1	1	1	1	1	1	1	1
2	3	3	2	2	2	3	3	2
3	3	3	3	3	3	3	3	4
4	2	3	2	3	1	4	3	1
2	2	2	2	2	2	2	2	2
3	3	4	3	3	3	3	3	3
3	3	3	3	3	3	3	3	3
3	2	3	3	2	2	2	3	3
2	2	2	2	2	2	2	2	2
3	3	3	3	3	3	3	3	3
2	2	3	3	2	2	3	2	1
3	3	3	3	3	3	3	3	3
1	3	3	2	3	2	3	3	2
1	1	1	1	1	1	1	1	1
2	3	3	4	3	3	3	4	3
3	4	4	4	3	3	4	3	4
2	2	3	3	3	3	3	3	2
4	4	3	3	3	3	3	3	3
3	1	3	2	2	2	2	1	1
2	3	4	2	3	3	2	3	2
3	3	3	4	4	4	4	4	4
4	4	4	4	4	4	4	4	4
3	3	3	3	3	3	3	3	3
3	3	3	3	3	3	3	3	3
3	3	3	3	3	3	3	3	3
3	3	3	3	3	3	3	3	3
2	2	2	2	2	2	2	2	2
1	1	1	1	1	1	1	1	1
4	4	4	4	4	4	4	4	4
2	3	4	3	2	2	3	3	3
1	1	1	1	1	1	1	1	1
2	3	3	2	2	2	2	2	3
4	4	4	4	4	4	4	4	4
3	3	3	3	3	3	3	3	3
3	3	3	3	3	3	3	3	3
3	2	2	2	2	2	2	2	2
4	4	4	4	4	4	4	4	4
3	3	3	3	3	3	3	3	2
1	3	4	4	4	4	3	3	3
1	2	2	1	1	1	1	1	1
1	1	1	1	1	1	1	1	1

1	1	1	1	1	1	1	1	1
2	4	3	4	2	3	3	3	3
1	3	2	3	2	2	2	2	1
2	3	2	3	2	3	2	3	2
1	1	1	1	1	1	1	1	1
2	2	3	1	1	2	2	2	2
3	3	3	3	2	2	3	3	2
3	1	3	3	3	3	3	3	1
3	3	3	3	3	3	3	3	3
3	3	3	3	3	3	3	3	3
1	1	1	1	1	1	1	1	1
3	1	2	3	3	2	2	3	3
1	4	4	4	4	4	4	4	2
2	2	2	2	2	2	2	2	2
2	3	3	3	3	3	3	3	1
3	3	3	3	3	3	3	3	3
3	3	3	3	3	3	3	3	3
2	3	3	3	3	3	3	3	3
3	3	3	3	3	3	3	3	1
3	4	3	1	3	3	3	3	1
1	1	1	1	1	1	1	1	1
2	2	2	2	2	2	2	2	2
2	2	2	2	2	2	2	2	2
2	3	1	1	1	1	2	1	1
4	3	4	3	3	3	3	3	3
2	2	2	2	2	2	2	2	2
3	3	3	3	3	3	3	3	3
2	4	2	2	2	2	3	3	1
4	4	4	2	2	3	3	4	1
2	2	2	2	2	1	1	1	1
3	4	3	2	3	4	3	3	1
3	3	3	3	3	3	3	3	3
4	4	4	4	4	4	3	4	4
2	3	2	2	2	2	2	2	2
3	3	3	3	3	3	3	3	3
2	4	3	2	3	3	3	3	1
2	2	2	2	2	2	2	2	2
2	2	2	2	2	2	2	2	2
2	3	3	3	3	3	3	3	3
3	3	3	3	3	3	3	3	3
3	3	1	1	1	1	1	1	2
3	3	3	3	3	3	3	3	2
3	3	3	3	3	3	3	3	2
3	3	3	3	3	3	3	3	3
1	1	1	1	1	1	1	1	1
3	3	3	3	3	3	3	3	3
4	4	4	4	4	4	4	4	4
1	1	1	1	1	1	1	1	1
1	1	3	2	1	1	1	1	1

3	3	3	3	3	3	3	3	3
2	1	2	1	1	1	1	1	1
1	4	3	4	2	2	2	3	2
4	1	4	3	3	3	3	4	4
3	3	3	3	3	3	3	3	3
3	3	2	2	3	2	3	2	1
2	2	2	2	2	2	3	3	1
3	3	3	3	3	3	3	3	1
2	3	3	3	3	2	2	2	2

B14
2
3
2
3
3
1
3
3
3
3
3
3
1
4
1
3
3
3
2
2
3
3
3
3
3
3
3
3
3
2
1
4
2
3
3
3
1
3
2
3
1
4
3
3
3
3

4
3
4
3
3
3
2
1
3
3
3
3
4
3
2
3
4
3
1
3
2
3
3
2
3
3
2
4
3
3
2
3
1
3
1
3
2
3
3
1
2
3
1
3
3
1
3
3
3

4
3
3
3
3
2
3
3
3
3
3
3
3
1
3
3
3
3
3
4
4
4
3
3
3
3
3
3
1
2
3
3
4
1
1
4
3
2
3
3
2
3
2
3
3
3
3
3
3

3
4
4
3
2
3
3
2
3
3
2
3
1
3
3
3
2
2
2
1
1
3
3
3
2
3
1
2
4
1
3
3
3
3
3
3
2
3
2
4
3
3
2
2
2
1
3
3
3

3
3
2
3
3
3
1
3
1
3
3
3
2
3
3
2
2
3
3
3
3
1
3
4
2
3
3
3
4
4
3
3
3
3
2
1
4
3
1
2
4
3
3
2
4
3
3
1
1

1
3
3
3
1
2
3
3
3
3
1
3
3
2
3
3
3
3
3
3
1
2
2
2
3
2
3
2
4
1
3
3
4
2
3
2
2
2
3
3
2
4
3
3
1
3
4
1
1

3
1
2
4
3
3
2
3
2
