# Supplementary material for: Can ChatGPT be trusted? Evaluating AI responses to oral health questions among pregnant Arabic-speaking women
Source: BMC Oral Health. 2025 Oct 10;25:1597. doi: 10.1186/s12903-025-06909-z (PMC12513007; doi:10.1186/s12903-025-06909-z)
Supplement: Supplementary file 2 — Supplementary Material 2. [file 12903_2025_6909_MOESM2_ESM.docx]

**Figure 1. Explaining ChatGPT to participants (English version).**


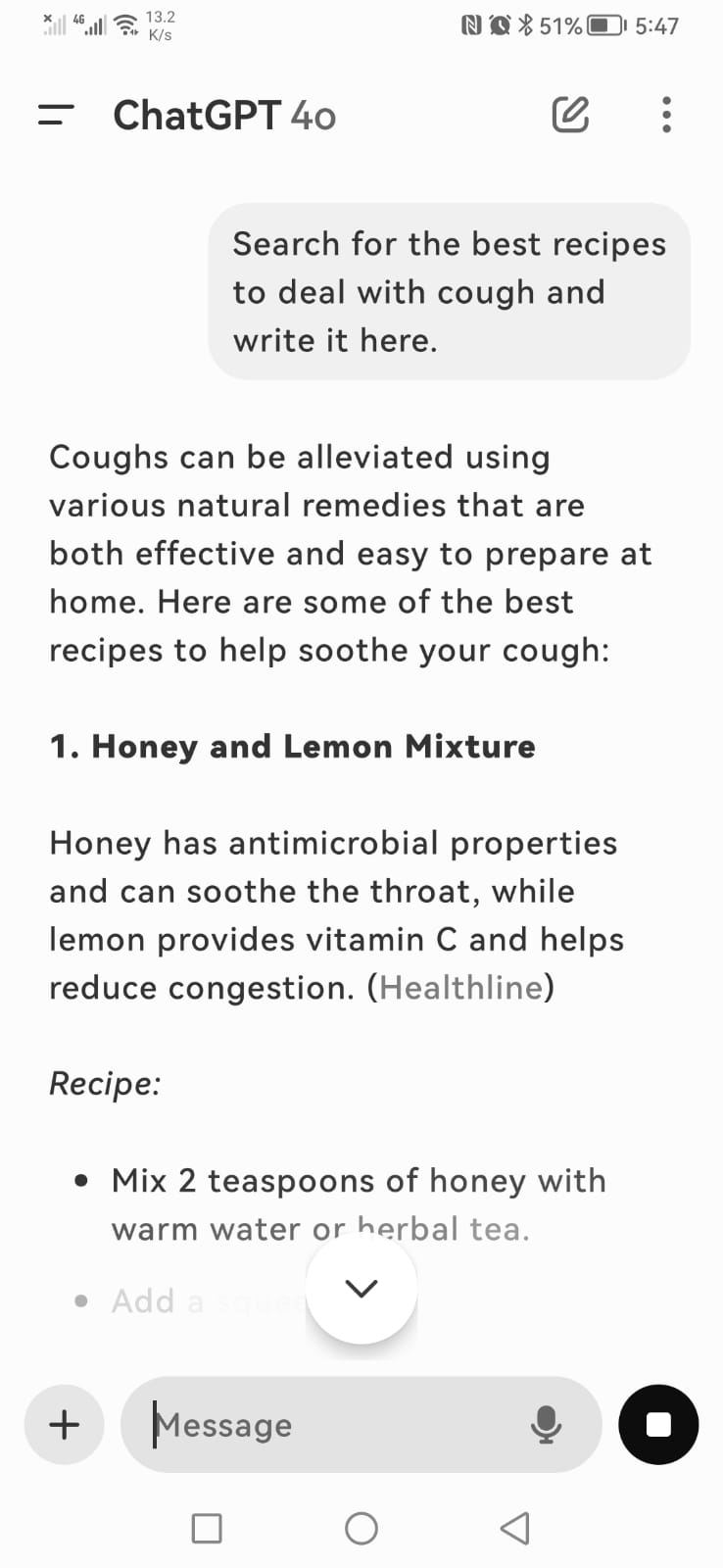


You can type any question in ChatGPT.

Here, ChatGPT responds to your request without human involvement, using artificial intelligence to fulfil the request or answer the question.

Example for a question
